# Improving the representation of adaptation in climate change impact models

**DOI:** 10.1007/s10113-018-1328-4

**Published:** 2018-04-13

**Authors:** Ian P. Holman, Calum Brown, Timothy R. Carter, Paula A. Harrison, Mark Rounsevell

**Affiliations:** 1 Cranfield Water Science Institute, Cranfield University, Vincent Building, Bedford, MK43 0AL UK; 2 Karlsruhe Institute of Technology, 82467 Garmisch-Partenkirchen, Germany; 3 Finnish Environment Institute (SYKE), FI-00251 Helsinki, Finland; 4 Centre for Ecology and Hydrology, Lancaster Environment Centre, Lancaster, LA1 4AP UK

**Keywords:** Adaptive capacity, Limits, Water, Land, Decision making, Integrated assessment

## Abstract

Climate change adaptation is a complex human process, framed by uncertainties and constraints, which is difficult to capture in existing assessment models. Attempts to improve model representations are hampered by a shortage of systematic descriptions of adaptation processes and their relevance to models. This paper reviews the scientific literature to investigate conceptualisations and models of climate change adaptation, and the ways in which representation of adaptation in models can be improved. The review shows that real-world adaptive responses can be differentiated along a number of dimensions including intent or purpose, timescale, spatial scale, beneficiaries and providers, type of action, and sector. However, models of climate change consequences for land use and water management currently provide poor coverage of these dimensions, instead modelling adaptation in an artificial and subjective manner. While different modelling approaches do capture distinct aspects of the adaptive process, they have done so in relative isolation, without producing improved unified representations. Furthermore, adaptation is often assumed to be objective, effective and consistent through time, with only a minority of models taking account of the human decisions underpinning the choice of adaptation measures (14%), the triggers that motivate actions (38%) or the time-lags and constraints that may limit their uptake and effectiveness (14%). No models included adaptation to take advantage of beneficial opportunities of climate change. Based on these insights, transferable recommendations are made on directions for future model development that may enhance realism within models, while also advancing our understanding of the processes and effectiveness of adaptation to a changing climate.

**Electronic supplementary material:**

The online version of this article (10.1007/s10113-018-1328-4) contains supplementary material, which is available to authorized users.

## Introduction

Climate change adaptation is an increasing necessity across human and natural systems, notwithstanding global efforts to reduce emissions, such as those stemming from the Paris Agreement. It can involve a broad range of short- to longer-term adjustments in social-ecological systems, the selection of which is influenced by interacting non-climatic changes, that seek to moderate harm or exploit beneficial opportunities arising from the actual or expected consequences of climate change (Moser and Ekstrom 2010). Adaptation is not easily reducible to theoretical or computational rules (Kandlikar and Risbey 2000; Crane et al. 2011), being framed by objective and subjective uncertainties about climate change impacts, vulnerabilities and the choices available to an adapting actor (Grothmann and Patt 2005; Travis and Huisenga 2013). Furthermore, adaptation is bounded by real-world constraints such as resource availability and human and institutional capacities (Berkhout 2012) and can be complicated by the spontaneous and natural adjustment of organisms to changing conditions. Therefore, the design and implementation of adaptation actions requires a holistic understanding of how individual and societal reactions to climate change interact with biophysical, social and economic processes. Without this understanding, there is a risk that adaptation planning may produce insufficient or even counterproductive results.

With such systemic complexity, modelling can be a valuable inductive and deductive tool for exploring adaptation strategies (Kelly et al. 2013). However, models that incorporate poorly grounded or weakly specified representations of adaptation may be more misleading than informative (Schneider et al. 2000; Irwin and Geoghegan 2001; Polhill et al. 2005). At the same time, model focus on the adaptive process should not obscure its dependencies on the particular impacts and responses involved. Hence, the representation of climate change adaptation (hereafter referred to as adaptation) within models requires careful consideration. Although adaptation is recognised as important in most climate change impact, adaptation and vulnerability (CCIAV) modelling studies, the climate-centric nature of many of the models used often precludes more integrated perspectives on decision-making and institutional processes (de Bremond et al. 2014). As a result, recent reviews (Brown et al. 2017; Olmstead 2014; Holman et al. 2012; Fisher-Vanden et al. 2011; Patt et al. 2010; Füssel 2010; Dickinson 2007) have found general weaknesses in the representation of adaptation in both sectoral and integrated assessment models, implying serious shortcomings in our ability to use models to develop robust adaptation strategies.

In this paper, we address three questions, with a focus on human systems. (1) What dimensions of climate change adaptation are identified as important by existing conceptualisations? (2) Which of these aspects are included in the current generation of models of land and water resource management? Therefore, (3) what (if any) improvements are necessary in these models’ representations of climate change adaptation? We answer these questions through a review of current conceptualisations of adaptation that allow us to derive criteria for the assessment of representations of adaptation in models. We apply these criteria to an extensive sample of models of the land and water sectors, and their applications, to ascertain the adaptation characteristics they currently include. In so doing, we recommend areas for focused improvement of such models to better support adaptation planning.

## Dimensions of adaptation and associated model requirements

Adaptation to climate change is the process by which human and natural systems respond to long-term changes in climatic conditions. As such, it is only meaningful to think of adaptation as a defined process at a highly abstracted level, below which numerous case-specificities become dominant. Here, we focus on adaptation in human systems, and in particular in those concerned with management of land and water resources. Even within this scope, a variety of different actions may be defined as adaptive in nature, despite sharing few if any specific characteristics. In order to account for this variety, the literature suggests that adaptation responses can be differentiated along a number of dimensions, implying key factors that models of adaptation must account for in some way (Smithers and Smit 1997). In the following, we focus on these primary dimensions of adaptation and their implications for model design.

### Form of adaptation

Adaptive responses can be triggered by a proactive decision process (planned adaptation) or occur spontaneously (autonomous adaptation) as an (often) indirect consequence of changes in natural or human systems (Smit et al. 2000; Füssel 2007; Tol et al. 2008; Japanese Ministry of Environment 2010; Berrang-Ford et al. 2011; IPCC 2014). This distinction has important implications for the timescales represented in models. While some adaptations may occur very rapidly (or are even effectively instantaneous, as with the spontaneous marginal adjustments in markets and individual behaviour modelled by some economists), many others require decades of planning and implementation. It is therefore important that models differentiate between adaptations with immediate and deferred benefits, and between those with different degrees of ‘lock-in’ that constrain future choices (Patt et al. 2010). At the same time, the potentially hierarchical or nested nature of these adaptations must also be accounted for, with planned adaptation allowing future autonomous adaptation (e.g. Noble et al 2014)). In modelling terms, autonomous (or spontaneous) and planned (or anticipatory) adaptation are differentiated by their timing in relation to climate-induced changes. Models must therefore be designed to allow adaptations in response to (subjectively understood) changes over wide time horizons, from the immediate past to the long-term anticipatable future.

### Trigger and objective

Adaptation may be triggered as a response to a potential (anticipated) or actual (reactive) impact threshold being breached. Commonly, such a threshold would be defined with respect to the range of impacts to be expected based on historical experience. Exceedance of a threshold may have positive or negative consequences, representing opportunities or threats. Given the multiplicity of potential adaptations, triggers and objectives (Tompkins et al. 2010), there is a widespread lack of clarity about how objectives determine the selection of adaptation options and their ultimate success or failure (Adger et al. 2005; Preston et al. 2011). Depending on the objectives in question, successful adaptation for one individual, group, organisation or government may have negative externalities and spillover effects at other spatial and temporal scales, increasing impacts on others or reducing their capacity to adapt (Adger et al. 2005; Füssel 2007). Therefore, it is also essential to consider the temporal and spatial extent of particular objectives and adaptations.

The complex nature of the triggers and objectives of adaptation make it imperative that models allow for a wide range of each of these, within which various synergies and trade-offs can occur (Rosenzweig and Tubiello 2007; Warren 2010; Berger and Troost 2014). Adaptation triggers can range from thresholds of impacts (e.g. economic losses, unacceptable number of people flooded; low river flows) to deviation from a certain level of production or service provision (e.g. meeting food demand or uninterrupted water supply). Furthermore, the exact triggers involved vary greatly from individual to governmental levels and are not always consistent with model assumptions concerning decision-making at these scales, which often focus on economics and single sectors in isolation. As a result, it is crucial that modelled triggers and objectives are explicitly identified and quantified so they can be related to those reported in reality (e.g. Tompkins et al. 2010).

### Spatial scale and sector

The scale-dependencies of adaptation may be simplified through the identification of especially important points or levels. Temporal factors lend themselves to some discretisation (for example, contrasting spontaneous with planned responses), while the spatial scale of adaptation (actions and decisions) can often be broken down into *national*, *regional* or *local* categories with different associated actors. There are, however, important exceptions where mismatches exist between the scale of adaptation and the scale at which it is modelled or at which its effects may be felt. These mismatches are particularly likely in supply chains and other transboundary settings (e.g. international waters or ecosystems) and in ecological, hydrological and other natural-systems units that do not correspond well to units of human decision-making, This poses considerable cross-scale and cross-jurisdictional challenges, and makes it essential that models deal clearly and carefully with issues of scale (Hewitson et al. 2014). Adaptations also often scale in terms of their sectoral focus, with some being highly sector-specific and others taking a holistic approach across multiple agencies, ministries and institutions so that adaptation planning becomes a cross-cutting issue (Bizikova et al. 2014). While the number of (interacting) scales of adaptation poses a serious challenge for modelling, the scope for specialisation in individual models is great, with complementarity of findings possible provided that issues of scale are dealt with clearly and rigorously.

### Actor and action

The identity of the adapting actor often implies a great deal about the form, timescale, objective and scale of the adaptation process. It can be useful to differentiate between adaptation measures that involve international bodies, governments (at multiple scales), private sector businesses, NGOs, formal and informal institutions, local communities and individuals (Keskitalo 2010; Juhola and Westerhoff 2011). These actors can undertake adaptation for the benefit of themselves or of others, with beneficiaries sometimes divided between private and public sectors (Adger et al. 2005; Tompkins and Eakin 2012). Furthermore, different types of action are available to these different actors, including physical (engineering, technological), investment or market-based, social and institutional adaptation measures (Adger et al. 2007). Many of these actions are difficult to model, which may explain the lack of diversity in actors and actions represented in models (Brown et al. 2017; Olmstead 2014; Holman et al. 2012; Füssel 2010). Nevertheless, the real-world range of actors involved in adaptation makes it important that models can include many types, with clear identification and justification of choices made.

### Constraints and effectiveness

Ultimately, the effectiveness of adaptation is its most important characteristic, and pre-assessment of this effectiveness is necessary to guide the selection or prioritisation of actions (Bizikova et al. 2014). However, there are many constraints that limit what an adaptation measure can deliver (Adger et al. 2007), often causing theoretical and realised effectiveness to diverge.

The capacity to adapt depends largely on contextual factors, which may include economic and natural resources, social networks, entitlements, institutions and governance, human resources, knowledge and technology (Schneider et al. 2000; Brooks et al. 2005; Moser and Ekstrom 2010). While these factors can become all-encompassing at a general level, they do have consistent characteristics across cases. *Economic constraints* are some of the greatest obstacles to real-world adaptation (World Bank 2008; Headwaters Economics 2012), both in their own right and in their implications for the use of (i.e. capacity to operate, finance and maintain) technology (Adger et al. 2007). More direct *technological constraints* can block the capacity to implement many adaptation options (Smit and Pilifosova 2003). *Knowledge constraints* can limit recognition of the need for adaptation and undermine the selection and operation of adaptation options (Milfont 2012; Tribbia and Moser 2008; Blennow and Persson 2009), while education and the provision of accessible climate information both facilitate more effective adaptation planning (Milfont 2012; Hamilton 2011).

*Physical and environmental constraints* include geographical barriers such as mountains, rivers and coastlines (Clark et al. 2011) or limits to land use change arising from water availability, soils, and human activity (Feeley and Silman 2010; Delgado et al. 2011; Shah 2009; Smit and Pilifosova 2003). *Social, ethical and cultural constraints* include preferences, norms, beliefs, perceptions of risk and self-efficacy, experience, shared knowledge and habitual behaviour (Adger et al. 2009; Nielsen and Reenberg 2010). Factors such as gender, age or religious belief can also influence the perception of risk (Lorenzoni et al. 2009; Sheridan 2007), the distribution of adaptive capacity and vulnerability in society (Jones and Boyd 2011; Bankston et al. 2010), and the perception of the utility of an adaptation measure.

*Effectiveness* can then be determined, in the context of these different constraints, according to the uptake of adaptive measures. Uptake can be evaluated not only through direct outcomes, but also through efficiencies of scale (Bizikova et al. 2014), although it is likely that the effectiveness of adaptation measures at a regional or national scale will be less than suggested by local bottom-up studies (Patt et al. 2010). Furthermore, the dependency of uptake on social and economic conditions, which may change under different socio-economic futures (e.g. O’Neill et al. 2017), adds temporal dynamism that models may struggle to account for (Adger et al. 2005).

## What adaptation characteristics are included in ‘climate change impact, adaptation and vulnerability’ (CCIAV) models?

Using the above adaptation characteristics as a framework, we reviewed the representation of adaptation within a range of modelling studies in the land and water sectors. The review was based on literature reported in international peer-reviewed journal articles using a number of independent searches in the Web of Science bibliographic database and snowballing (of journal articles and grey literature reports published by funding bodies) to identify models dealing with adaptation in distinct ways and to ensure representative coverage of CCIAV studies and models in these sectors (see search terms and references within the Supplementary Material). The review was not intended to be all-encompassing, but examined a cross-section of relevant models covering different modelling approaches, research questions, adaptation measures and spatial and temporal scales. For the water sector, these spanned models representing river channels, catchments/river basins and water resource systems; and study foci on management of flood risk, water resources and quality, water demand and infrastructure (abstraction, distribution and supply). For the land sector, a range of model types (including Agent-based, statistical allocation, partial equilibrium models etc) operating at scales from farm to global and representing all major forms of productive human land use were included, with particular foci on agriculture, forestry, and cross-sectoral dynamics. . We sought to evaluate each model’s treatment of the above dimensions of adaptation both statically and dynamically by assessing the following criteria:

- The spatial, temporal and geographical scales over which the model was applied;
- The extent to which adaptation, if included explicitly, was represented objectively on the basis of exceedance of defined realistic impact triggers (as opposed to being implemented by the modeller through the modification of input parameters following some arbitrary impact change);
- The type (autonomous or planned) and timing (reactive or anticipatory) of adaptation included;
- The actors involved—whether individual, institutional, governmental, aggregate or unspecified;
- How adaptation effectiveness is modelled, especially with respect to timelags, constraints (biophysical, social, human, financial or manufactured) and rates or processes of uptake;
- Any temporal (scenario-based) variability in modelled adaptation.

**Fig. 1:**
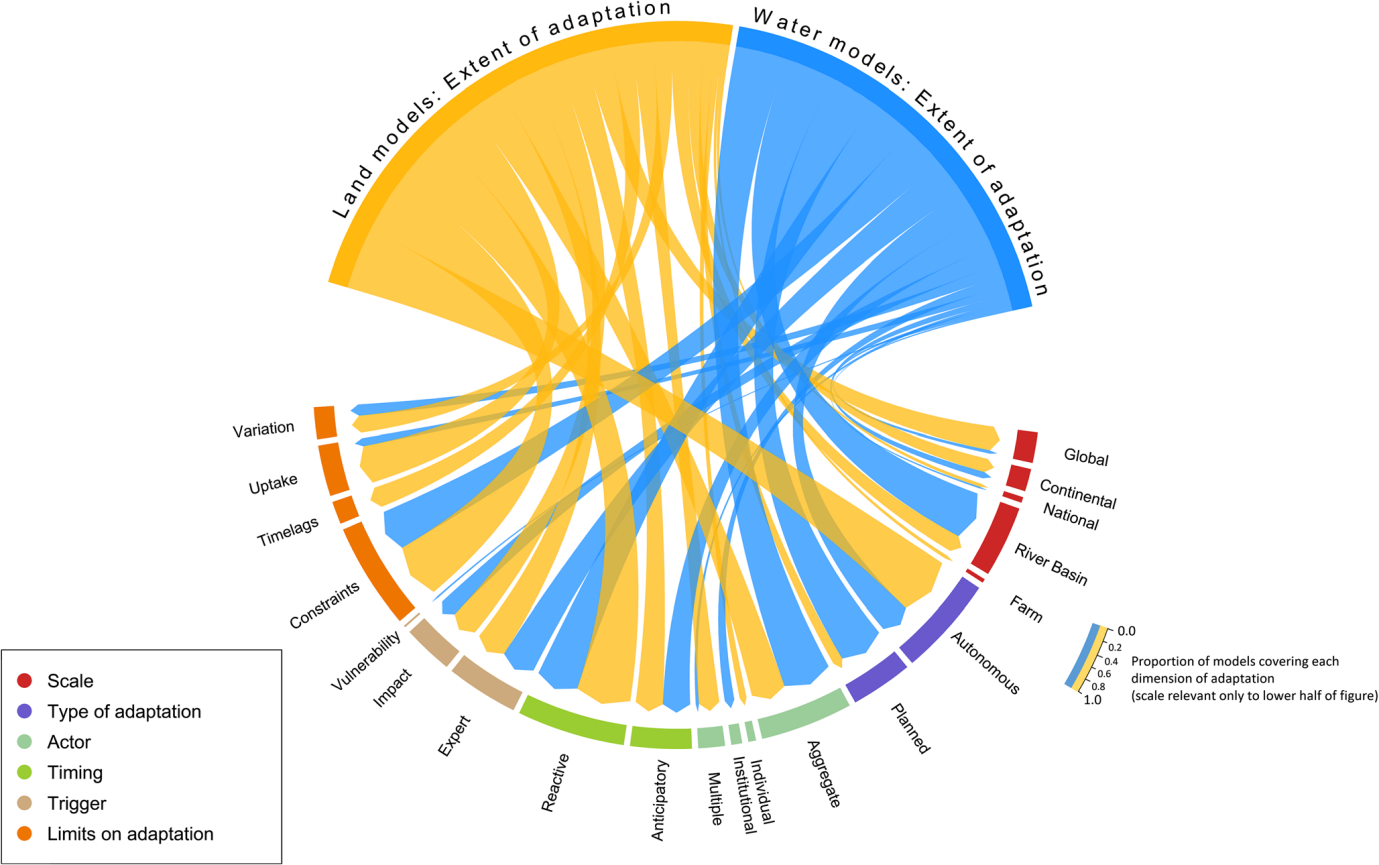
Relative coverage of dimensions of adaptation (lower half of figure) by land and water sector models (upper half of figure). The thickness of lines linking the model sector to the adaptation dimensions scales with the proportion of models of that sector that include each dimension; therefore, arrow tips in the lower half of the figure are all directly comparable. Because the individual dimensions are not mutually exclusive, the radial extent of each sector in the upper half of the figure provides a relative measure of the coverage of models within that sector rather than an absolute measure of the number of models

In total, 18 land use allocation models applied in 20 studies and 22 water management models applied in 42 studies were reviewed. The results are summarised in Fig. 1, with further details in Table SM1 for land use allocation models and Table SM2 for water management models.

In general, representation of adaptation characteristics was found to vary between models of the land and water sectors (Fig. 1). In land sector models, adaptation was more often included explicitly (in 77% of models) and more comprehensively, but only triggered by very specific climatic impacts that threatened equilibrium of supply and demand or an assumed achievement of profit-maximisation. These triggers consistently relate to underlying model assumptions (e.g. about economic optimisation and market equilibrium) rather than observed triggers of real-world adaptation. Adaptations in response to multiple climate impacts and their non-market effects are therefore underexplored. In the water sector, adaptation was less often included explicitly (in 21% of models), and its triggers were even less clearly defined, with adaptation actions and timings usually being determined subjectively by the modeller (due to the prevailing physical, rather than economic, focus of many water sector models). While adaptation triggers based on either an objective function (e.g. water supply-demand balance) or arbitrary changes in state indicators (e.g. river flow, crop yields) are evident with most of the water models (Table SM2; demonstrating the importance of the modeller in setting objective adaptation triggers), physically-based river basin hydrological models (e.g. SWIM, SWAT etc) are currently less-suited for implementing objective trigger-based adaptation compared to the system optimisation (e.g. CALVIN etc) and water accounting models (e.g WEAP).

For both sectors, adaptation was commonly represented as the result of autonomous (unplanned) decisions (45% of models) by aggregate or undefined decision-making entities (71% of models). This was especially true at larger (national to global) scales, where few models investigated either the combination of multiple adaptations by individual actors, or explicit adaptation by governmental actors. At smaller scales (farm, river basin etc.), individual-level adaptation has been considered, especially by agent-based land use models, and this was also the only context in which model development was found to have included meaningful stakeholder (actor and/or decision-maker) participation.

**Fig. 2:**
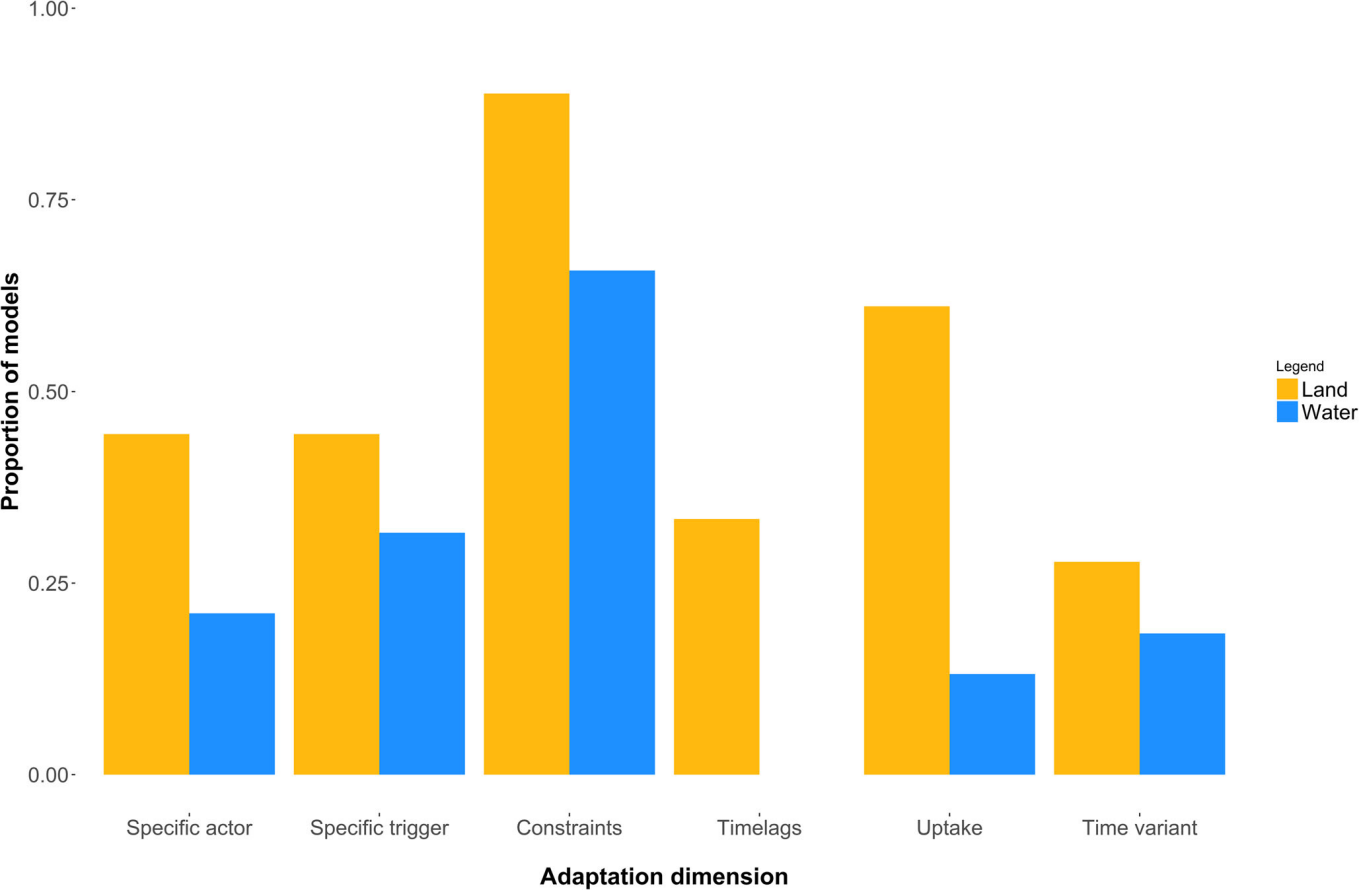
The proportion of reviewed land and water sector models incorporating different adaptation dimensions

Once made, the implementation of adaptation decisions was largely unconstrained, with 27% of models (1% of land sector models and 34% of water sector models) implementing no constraints, timelags or uptake limits on adaptation whatsoever, and only 11% of models (33% of land sector models and no water sector models) implementing all three (Fig. 2). This reveals a widespread working assumption that modelled adaptation options were immediately and comprehensively available to all actors. Where socio-economic conditions affected adaptation (mainly in land sector models), they often did so only to the extent that such relationships were encapsulated in statistical trends based on previously observed changes (e.g. where historical effects of economic growth on land use change were used to calibrate economic constraints on adaptation). Furthermore, the treatment of climatic and socio-economic changes was often limited, with one or two scenarios being used in the majority of cases. Otherwise, adaptations that satisfied some biophysical and financial criteria were almost invariably modelled as having no further spatial or temporal dependencies, with the notable exception of some land sector (mainly agent-based) models that included facilitation or limitation of adaptation on the basis of social networks, neighbourhood effects or individual agent characteristics (Acosta-Michlik and Espaldon 2008; Baranzelli et al. 2014).

Temporal variations in the nature of adaptation options and decisions were only included in a few models (27% of land system models and 18% of water system models—Fig. 2) and generally via changes in socio-economic or productive context rather than the adaptive process itself. However, these changes were tightly constrained, generally being linear in nature up to some subjective maximum, and applied uniformly across the population of modelled actors. However, the application of most models to artificially isolated timeslices (which disconnect the baseline and a future time period, rather than simulating the full transient behaviour from the beginning of the baseline period through to the end of the future period) make these limitations hard to avoid, as well as eroding the distinction between anticipatory and reactive adaptation.

Notwithstanding some commonality of approach to adaptation modelling, a range of model types has been applied across a range of scales in both land and water sectors. These model types include a variety of basic assumptions and architectures, providing differing perspectives on adaptation. Nevertheless, hybrid approaches appear to be extremely rare, as are applications of multiple models to the same case study, which would allow exploration of uncertainties as well as potentially integrated approaches. Overall, therefore, the models we reviewed showed strong specialisation, individually and collectively, in terms of sectoral and process focus, leaving interactions between these highly under-represented.

## Discussion

The ability of models to improve inductive understanding and deductive projection of climate change adaptation is of considerable relevance to adaptation planning and policy processes, such as National Adaptation planning, risk assessments and numerous national and international legislation and policies. However, our findings suggest that CCIAV models may not yet be equal to the challenge. We find a clear and substantial distinction between the extensive, available empirical knowledge about adaptation processes and their lack of representation in models of the land and water sectors. In general, models tend to neglect significant aspects of adaptation in favour of detailed representations of specific issues (such as changes in agricultural land area as yields change, or changing sectoral water demand or infrastructural-based supply as availability changes). A prevalence of simplistic, over-optimistic approaches (due to omissions of many of the important characteristics of actual adaptation) to simulating the potential for adaptation to reduce impacts and vulnerabilities or exploit benefits associated with climate change means that many CCIAV studies produce outcomes that cannot meaningfully inform adaptation planning.

The fundamentally holistic nature of adaptation means that research needs to span a broad spectrum of issues that are traditionally studied by distinct scientific disciplines. However, while models have made significant progress in representing processes within systems or sectors, when applied in isolation they are not sufficient to address the complexity of adaptation decisions, which must account for feedbacks and interdependencies between those systems or sectors (Harrison et al. 2016). This shortcoming is exacerbated by the lack of links and multi-scale comparisons between the models we review here—existing comparisons have considered only large scales such as European and global (e.g. Alexander et al. 2016a). At global scales, integrated assessment models that are ideally placed to trace cross-sectoral effects of climate change and adaptations, and to incorporate local responses to global drivers, rarely do so (Sohngen et al. 2001; Rose 2014). Meanwhile, local-scale models of specific adaptations or social processes neglect rigorous explorations of external pressures (Brown et al. 2017); a particular problem with agent-based models that become more difficult to implement and interpret as their complexity increases. In both cases, recent advances towards model applications at intermediate (e.g. national-continental) scales hold promise for the unification of existing areas of knowledge (e.g. van Asselen and Verburg 2013; Magliocca 2015; Blanco et al. 2017), as well as allowing modelling at scales more congruent with real-world adaptation processes and effects. In particular, the inclusion of specific adaptation actors and land or water systems that operate across regional scales is a necessary step forward.

Similarly, there has been a notable absence of cross-sectoral or regional interactions in models of adaptation to date (Harrison et al. 2016). We find several models that build on the obvious links between agriculture and water resources (e.g. Hayashi et al. 2013; Girard et al. 2015a, b; Vaghefi et al. 2015; Mango et al. 2011; Arnold et al. 2015), but extremely important teleconnections and links between pastoral and arable agriculture; agriculture, forestry and urban development; and levels of land management intensity are insufficiently represented (Seto et al. 2012; Meyfroidt et al. 2013; Rosa et al. 2014; Rose 2014; Brown et al. 2017). This precludes essential understanding of synergies, trade-offs and other implications between adaptations in different areas and sectors, as well as the ways in which these may mutually alter the long-term impacts of climate change and scope for adaptation (Warren 2010). This deficiency also extends to feedbacks between adaptive and mitigative actions, which are generally modelled separately (Brown et al. 2017).

Of course, many of the models currently used to simulate climate change adaptation were not originally developed for this purpose. Furthermore, differences in basic model assumptions are partly responsible for the disjointed nature of modelled adaptation. For instance, the temporal development of demand and supply in general equilibrium models is quite distinct from the decision-based dynamics of agent-based models, reflecting the absence of coherent theories of land system change and sectoral water demand. Linking the two requires more than an alignment of model parameters if meaningful results are to be produced. This problem is even more challenging for the integration of knowledge derived from anthropology and social sciences about socio-cultural perspectives on climate impacts and adaptation options. The widespread lack of participatory and inter-disciplinary modelling approaches is a notable shortcoming in this context.

There is also a clear lack of coverage in some important areas. Major gaps include climate anticipation and demand-side adaptations such as changing levels of meat demand, and land and water usage in tropical grasslands, rangelands and forests (Rosa et al. 2014; Rose 2014; Alexander et al. 2016b). Possibly more important still are climate-dependencies in trade systems and supply chains that support production across remote locations, and which are rarely, if ever, modelled (Levermann 2014). All of these omissions introduce substantial uncertainties and inaccuracies, because they mean that important links and feedbacks cannot be represented.

Equally significant is the inadequate exploration of the effects of socio-economic or climatic uncertainty on adaptation processes and decisions; a common shortcoming amongst the models we reviewed. Scenarios provide very different contexts in which to identify the ‘best’ adaptation options and will impose very different economic, governance and social constraints on implementation and effectiveness. Despite high-profile development of exploratory socio-economic scenarios including the recent Shared Socio-economic Pathways (SSPs: O’Neill et al. 2017), only a small number of scenarios are applied in most modelling studies. This gives a highly conditional and unrepresentative impression of adaptation options, especially where it enables apparent optimisation based on predictability of future impacts (Pindyck 2017). While a small number of studies allow scenario constraints to condition adaptation over time (e.g. Murray-Rust et al. 2014; Brown et al. 2016; Steinbuks and Hertel 2016), most are likely to overestimate the amount of adaptation that will occur within many scenarios and therefore also overestimate the benefits obtained from adaptation (Patt et al. 2010; Harrison et al. 2016).

Finally, many models either directly or indirectly assume optimal adaptation, or adaptation that continues previously observed trends; a particular issue for statistical models that implicitly, but inappropriately, assume a continuation of previous adaptation trends (Rose 2014; Brown et al. 2017). As Patt et al. (2010) argue, ‘optimal adaptation is not a good representation of the past, and probably is not a good representation of the future, because social and political constraints get in the way’. Furthermore, such adaptation does not take account of the possibility of maladaptation (where actions undertaken either directly to address changing climate or independently to address other challenges, may accentuate adverse climate-related outcomes), of failure to adapt due to the obscuration of climatic trends (e.g. by weather variability), of novel (potentially highly beneficial) adaptations, or of adaptations to unexpected regime shifts in socio-ecological systems (Schneider et al. 2000; McLeman and Smit 2006; Filatova et al. 2016).

## Conclusions and ways forward

Our review of a broad range of land- and water-based models has demonstrated that the treatment of adaptation is currently fragmented and often simplistic. Land use models generally provide a better representation of economic and behavioural constraints on adaptation, though without either fully capitalising on the advantages of behavioural modelling or fully overcoming its challenges for parameterisation, calibration and validation. Few of the other non-behavioural models reviewed are able to explicitly and objectively simulate adaptation and its consequences, with particular shortcomings apparent in the (1) dominance of autonomous decisions made by aggregate or undefined decision-making entities, which are (2) based on subjective decisions rather than explicit impact-based threshold exceedance, (3) limited to an artificially constrained range of options, (4) rarely subject to spatio-temporal variation or constraints even under extreme scenarios, and (5) poorly integrated with other sectors or scales.

Nevertheless, it is also clear that many individual models and their applications provide examples of good practice upon which to build. We therefore offer our suggestions for how the modelling community should move forward with improving the simulation of climate change adaptation, reflecting the value to be gained from integrating a bottom-up understanding of adaptation as a socio-ecological process into computational models:

- Move beyond unrealistic ‘business as usual’ scenarios that extrapolate the recent past without taking account of the numerous disruptive technologies, social movements or behaviours that might occur (O’Neill et al. 2017).
- Embrace scenario uncertainty rather than trying to identify predictable or ‘most likely’ scenarios, while a single optimum model solution to adaptation might appear attractive, relying on an apparent ability to predict future risks or to foresee the eventual outcomes of decisions is an inappropriate and unreliable paradigm (Lempert and Collins 2007).
- Analyse adaptation responses via uncertainty-based frameworks such as robust decision-making and adaptive planning (e.g. Gleeson et al. 2011; Holman and Trawick 2011) or precautionary cost-benefit (Beven 2011) to identify low or no-regret options.
- Work with stakeholders and decision-makers to better understand the triggers and goals of adaptation policies and measures.
- Reflect on the importance of extreme events in driving adaptation (Berrang-Ford et al. 2011).
- Include adaptations that take advantage of climate change rather than simply defensively respond to negative impacts (Berrang-Ford et al. 2011).
- Embed human and social behaviours and constraints within models, either through integrating agent-based models with process-based models or through structured approaches to constrain model input changes that reflect time-varying scenario-specific settings.
- Account for the full cost of adaptation, in terms of the type and the amount that can occur, reflecting the financial constraints on adaptation.
- Allow for positive and negative effects on resource availability: while implementing some types of adaptation can use up the availability of some capital types (e.g. financial and manufactured), the implementation of some people-based adaptation measures can increase human and social capital.
- Recognise the importance of cross-sectoral interactions and dependencies that can enhance the overall benefits of adaptation or lead to trade-offs and unintended consequences (Harrison et al. 2016).
- Focus on capturing the spatial and temporal dependencies in adaptation options, capacities, decisions and effects.
- Relax the (artificial) distinction between autonomous and planned adaptation, which can obscure the roles of the state and society in producing the circumstances in which autonomous adaptations can or cannot occur (Adger et al. 2003).
- Adopt a diversity of approaches, in a diversity of sectors and scales, to build up a picture of adaptation. ‘Open recognition of the limited set of assumptions contained in any one study of adaptation demands that authors clearly note that each individual study can represent only a fraction of plausible outcomes’ (Schneider et al. 2000) .
- Recognise that adaptation can be hierarchical, with adaptation decisions at one level (of governance or scale) potentially leveraging or otherwise influencing adaptation decisions at other levels.
- Account for external adaptations occurring (or required) outside a given jurisdiction, which may influence domestic outcomes or adaptation requirements.
- Consider adaptation alongside mitigation within an integrated climate policy framework.

While the above represents an Agenda for improvement within ‘next generation’ CCIAV models, the review has also demonstrated that there is an urgent need for short-term focused improvements in the treatment of adaptation in existing models. In particular, a movement away from subjective and opaque adaptation triggers and often arbitrary or modeller-defined changes to model inputs, towards objective and transparent criteria for implementing adaptation within the models, realistic treatment of timelags in the parameterisation of different measures and transparent assumptions regarding the scenario-specific constraints on the adaptation parameterisation. Despite the Paris Agreement, the continuing need to adapt to climate change behoves the CCIAV modelling community to provide the realistic assessments needed to support improved decision-making.

## Electronic supplementary material

ESM 1 (DOCX 76 kb)
