# Supplementary material for: Improving the representation of adaptation in climate change impact models
Source: Reg Environ Change. 2018 Apr 13;19(3):711–21. doi: 10.1007/s10113-018-1328-4 (PMC6418063; doi:10.1007/s10113-018-1328-4)
Supplement: Supplementary file 1 — (DOCX 76 kb) [file 10113_2018_1328_MOESM1_ESM.docx]

**Improving the representation of adaptation in climate change impact models**

Ian P Holman^1^, Calum Brown Timothy R Carter, Paula A Harrison, Mark Rounsevell

^1^ Cranfield Water Science Institute, Cranfield University, Bedford MK43 0AL, UK

E-mail: [i.holman@cranfield.ac.uk](mailto:i.holman@cranfield.ac.uk)

**Search terms used**

(‘land use change’ or ‘land use’ or ‘land cover’ or ‘land based’ or ‘land management’ or ‘agriculture’ or ‘agricultural’ or ‘forest’) and (‘model’ or ‘modelling’ or ‘modeling’) [with and without] (‘climate’ or ‘climate change’) or ‘adaptation’

(‘climate change’ and ‘[model name]’) where [model name= = Aquator, HEC, HBV, Infoworks, LisFlood, MIKE, SWAP, SWAT, SWIM, VIC, WaSim, WaterGAP, WEAP, WMS]

‘water’ and ‘model’ and ‘climate change’ and ‘agent’

‘water demand’ and ‘model’ and ‘climate change’ and ‘adapt’

**Table SM1 Analysis of the Land use allocation models**

| Model (and references) | Scale of application | Model type | Adaptation within model? | |  | Adaptation elements included within model applications | | | | | |
| --- | --- | --- | --- | --- | --- | --- | --- | --- | --- | --- | --- |
|  |  |  | Explicitly represented? | Adaptation type? | Actors | Trigger | | Effectiveness within scenario | | | Over time |
|  | G / C/ N / RB / F^[[1]](#footnote-1)^ |  | (Yes / No) | A / P ^[[2]](#footnote-2)^ | Ind, Inst, G, A^[[3]](#footnote-3)^ | Timing (A / R)^[[4]](#footnote-4)^ | Type  (E, I, V)^[[5]](#footnote-5)^ | Constraints  (B / S / H / F / M)^[[6]](#footnote-6)^ | Timelags | Uptake | Time-variant? |
| CRAFTY [*Murray-Rust et al.*, 2014; *Blanco et al.*, 2017] | C/N | ABM | Y | A (Land use change & intensification/ extensification) | Ind | R | E | B, S, H, F, M | Diffusion through social networks | Limited by agent characteristics | Only as function of socio-economic scenarios |
| MPMAS [*Arnold et al.*, 2015] | RB | ABM-hydrological linked | Y | A (resources, management practice, investment, water usage) | Ind, Inst (tradeable water rights) | A, R | E (V) (expected or actual shortfall of water relative to crop requirements) | B, S, H, F, M | Some adaptations not immediately available; constraints on uptake | Some economic constraints (ability to invest) | No |
| Unnamed [*Berman et al.*, 2004] | F | ABM | Y | A (migration, hunting activities, household formation, resource sharing) | Ind, Inst | R | E (I) | B, S, H, F | Only through model timesteps (5 years) | Constrained by agent characteristics | Population characteristics change over time, affecting adaptation options and uptake |
| LandClim [*Yousefpour et al.*, 2015] | RB (Forest Landscape) | Microsimulation | Y | A (management intensity/ objective) | Ind | A / R (depending on type of actor) | I/V (adapt to maximise expected production levels or observed outputs) | B | Limited number of decision points – long-term forest management | Constrained by actor type | Yes: changes in expectations of climate change |
| CLUMondo [*van Asselen and Verburg*, 2013] | G | Allocation (statistical) | N (only in response to demand changes, but equivalent process to climate/productivity change) | A (land use change and intensification/ extensification) | A | R | I (adapt to ensure supply=demand for food and urban areas) | B, others encapsulated in empirically-based statistical relationships | No | Conversion costs; neighbourhood influence | No |
| EcoChange [*Dendoncker et al.*, 2006] | RB (equivalent scale) | Statistical allocation (autoregressive) | Y | A (land use and cover change) | A | R | I (land use area = scenario-based area demand for each land use | Only as encapsulated in statistical probabilities of land use occurence | No | Land use hierarchy constrains change; neighbourhood influence | No |
| MAgPIE [*Popp et al.*, 2014] | G | Optimization / Partial Equilibrium | Y | A (land use change, particularly forest/agriculture) | A | A, R (anticipatory mitigation policies affect reactive adaptation) | I (food & livestock supply=demand) | B, S, F | 10-year timesteps | Conversion costs, mitigation policies | No |
| PLUM [*Engström et al.*, 2016a, 2016b] | G | Statistical / rule-based | Y | A (agricultural land use) | A | R | I (ensuring food supply = demand) | B, S, F | No | Wherever appropriate | No |
| Unnamed [*Acosta-Michlik and Espaldon*, 2008] | RB (municipality) | ABM | Y | A (consumption, production, cooperation; adaptation schemes) | Ind, G (adaptation policy scenarios) | R, A (policy scenarios) | E (I, V) (Expert judgement of when impacts of vulnerability prompt adaptation) | B, S, H, F | Temporal effects of social networks | Limited by social networks and individual characteristics | No |
| CAPS [*Meiyappan et al.*, 2014] | G | Optimization, allocation (statistical) | N (land use responds to historical climatic drivers but not explicit adaptation) | A (autonomous responses based on profit maximisation) | A | R | E/I (Profit maximisation) | B, S, F | Spatial and temporal autocorrelation included | | No |
| GLOBIOM [*Havlík et al.*, 2014] | G | Partial equilibrium; allocation | N (CC not directly modelled) | A (land use change) | A, G (Mitigation policies) | R (A for mitigation) | E (global optimisation with constraint) | B, F (conversion cost) | 10-year timesteps | Not otherwise constrained | No |
| MIT IGSM [*Prinn et al.*, 1999; *Reilly et al.*, 2007] | G | CGE | Y | A (Economically-driven changes in production resulting from CC impacts on yields) | A | R | E/I (equilibrium model; adjusts to negate impacts) | B, F | No | No | No |
| Unnamed [*Mu and McCarl*, 2011] | N | Econometric (statistical) | Y | A (land use change between arable, pastoral and others) | A (aggregate changes in land use) | R | I (based on observed changes) | None (except those contained in statistical relationships) | No | No | No |
| LUMP [*Lavalle et al.*, 2013] | C | General/static partial equilibrium | N (climate change not modelled) | P (models effects of CC strategies, mainly mitigation) | G, A (Gov. policies, aggregate LUC) | A | No trigger; modeller-defined | B, F (possible) | No | No | No |
| LUISA [*Baranzelli et al.*, 2014] | C | Statistical/ rule-based | Y | A (land use change) | A | R | I (yields) | B, S, F, M | Yes (neighbourhood effect) | Yes (neighbourhood effect) | Yes |
| FABLE [*Steinbuks and Hertel*, 2016] | G | Partial equilibrium | Y | A, P (land use change, deforestation change) | A (representative agent) | R, A | I (E) (welfare maximisation) | B, S, F | No | No | No (scenario conditions change but adaptation processes constant) |
| CLIMSAVE IAP [*Harrison et al.*, 2015] | C | Integrated model (meta-models) | Y | A, P (land use change, intensification/ extensification) | A | R | E (I, V) | B, S, F, M (capitals) | No | No | Yes -Socio-economic scenario effects on capitals |
| RULEX [*Bakker et al.*, 2014, 2015] | RB | ABM | Y (land exchange as adaptation) | A (land use and ownership change) | I, Inst | R | E (informed by survey) | F | No | No | No |

**References for Table SM1**

Acosta-Michlik, L., and V. Espaldon (2008), Assessing vulnerability of selected farming communities in the Philippines based on a behavioural model of agent’s adaptation to global environmental change, *Glob. Environ. Chang.*, *18*(4), 554–563, doi:10.1016/j.gloenvcha.2008.08.006.

Arnold, R. T., C. Troost, and T. Berger (2015), Quantifying the economic importance of irrigation water reuse in a Chilean watershed using an integrated agent-based model, *Water Resour. Res.*, *51*(1), 648–668, doi:10.1002/2014WR015382.

van Asselen, S., and P. H. Verburg (2013), Land cover change or land-use intensification: simulating land system change with a global-scale land change model, *Glob. Chang. Biol.*, *19*(12), 3648–3667, doi:10.1111/gcb.12331.

Bakker, M., S. J. Alam, J. van Dijk, M. Rounsevell, T. Spek, and A. van den Brink (2015), The feasibility of implementing an ecological network in The Netherlands under conditions of global change, *Landsc. Ecol.*, *30*(5), 791–804, doi:10.1007/s10980-014-0145-5.

Bakker, M. M., S. J. Alam, J. van Dijk, and M. D. A. Rounsevell (2014), Land-use change arising from rural land exchange: an agent-based simulation model, *Landsc. Ecol.*, *30*(2), 273–286, doi:10.1007/s10980-014-0116-x.

Baranzelli, C., C. Jacobs-Crisioni, F. Batista, and C. Castillo (2014), The reference scenario in the LUISA platform—updated configuration 2014 towards a common baseline scenario for EC impact assessment procedures, *Publications Office of the Europan Union*.

Berman, M., C. Nicolson, G. Kofinas, J. Tetlichi, and S. Martin (2004), Adaptation and Sustainability in a Small Arctic Community: Results of an Agent-Based Simulation Model, *Arctic*, *57*(4), 401–414, doi:10.2307/40512643.

Blanco, V., C. Brown, S. Holzhauer, G. Vulturius, and M. D. A. Rounsevell (2017), The importance of socio-ecological system dynamics in understanding adaptation to global change in the forestry sector, *J. Environ. Manage.*, *196*, 36–47, doi:10.1016/j.jenvman.2017.02.066.

Dendoncker, N., P. Bogaert, and M. Rounsevell (2006), A statistical method to downscale aggregated land use data and scenarios, *J. Land Use Sci.*, *1*(2–4), 63–82, doi:10.1080/17474230601058302.

Engström, K., M. D. A. Rounsevell, D. Murray-Rust, C. Hardacre, P. Alexander, X. Cui, P. I. Palmer, and A. Arneth (2016a), Applying Occam’s razor to global agricultural land use change, *Environ. Model. Softw.*, *75*, 212–229, doi:10.1016/j.envsoft.2015.10.015.

Engström, K., S. Olin, M. D. A. Rounsevell, S. Brogaard, D. P. van Vuuren, P. Alexander, D. Murray-Rust, and A. Arneth (2016b), Assessing uncertainties in global cropland futures using a conditional probabilistic modelling framework, *Earth Syst. Dyn. Discuss.*, *0*, 1–33, doi:10.5194/esd-2016-7.

Harrison, P. A., I. P. Holman, and P. M. Berry (2015), Assessing cross-sectoral climate change impacts, vulnerability and adaptation: an introduction to the CLIMSAVE project, *Clim. Change*, *128*(3–4), 153–167, doi:10.1007/s10584-015-1324-3.

Havlík, P. et al. (2014), Climate change mitigation through livestock system transitions., *Proc. Natl. Acad. Sci. U. S. A.*, *111*(10), 3709–14, doi:10.1073/pnas.1308044111.

Lavalle, C., S. Mubareka, C. Perpiña Castillo, C. Jacobs-Crisioni, C. Baranzelli, F. Batista Silva, and I. Vandecasteele (2013), *Configuration of a reference scenario for the land use modelling platform*.

Meiyappan, P., M. Dalton, B. C. O’Neill, and A. K. Jain (2014), Spatial modeling of agricultural land use change at global scale, *Ecol. Modell.*, *291*, 152–174, doi:10.1016/j.ecolmodel.2014.07.027.

Mu, J. H., and B. A. McCarl (2011), *Adaptation to Climate Change: Land Use and Livestock Management Change in the U.S.* Southern Agricultural Economics Association Annual Meeting, Corpus Christi, TX, February 5-8, 2011*.*

Murray-Rust, D., C. Brown, J. van Vliet, S. J. Alam, D. T. Robinson, P. H. Verburg, and M. Rounsevell (2014), Combining agent functional types, capitals and services to model land use dynamics, *Environ. Model. Softw.*, *59*, 187–201, doi:10.1016/j.envsoft.2014.05.019.

Popp, A., F. Humpenöder, I. Weindl, and B. Bodirsky (2014), Land-use protection for climate change mitigation, *Clim. Chang.*

Prinn, R. et al. (1999), Integrated Global System Model for Climate Policy Assessment: Feedbacks and Sensitivity Studies, *Clim. Change*, *41*(3/4), 469–546, doi:10.1023/A:1005326126726.

Reilly, J., S. Paltsev, B. Felzer, X. Wang, D. Kicklighter, J. Melillo, R. Prinn, M. Sarofim, A. Sokolov, and C. Wang (2007), Global economic effects of changes in crops, pasture, and forests due to changing climate, carbon dioxide, and ozone, *Energy Policy*, *35*(11), 5370–5383, doi:10.1016/j.enpol.2006.01.040.

Steinbuks, J., and T. W. Hertel (2016), Confronting the Food–Energy–Environment Trilemma: Global Land Use in the Long Run, *Environ. Resour. Econ.*, *63*(3), 545–570, doi:10.1007/s10640-014-9848-y.

Yousefpour, R., M. Didion, J. B. Jacobsen, H. Meilby, G. M. Hengeveld, M.-J. Schelhaas, and B. J. Thorsen (2015), Modelling of adaptation to climate change and decision-makers behaviours for the Veluwe forest area in the Netherlands, *For. Policy Econ.*, *54*, 1–10, doi:10.1016/j.forpol.2015.02.002.

**Table SM2 Analysis of the water-based models**

| Model (and references) | Scale of application | Model type | Adaptation within model? | |  | Adaptation elements included within model applications | | | | | |
| --- | --- | --- | --- | --- | --- | --- | --- | --- | --- | --- | --- |
|  |  |  | Explicitly represented? | Adaptation type? | Actors | Trigger | | Effectiveness within scenario | | | Over time |
|  | G / C/ N / RB / F^[[7]](#footnote-7)^ |  | (Yes / No) | A / P ^[[8]](#footnote-8)^ | Ind, Inst, G, A^[[9]](#footnote-9)^ | Timing (A / R)^[[10]](#footnote-10)^ | Type  (E, I, V)^[[11]](#footnote-11)^ | Constraints  (B / S / H / F / M)^[[12]](#footnote-12)^ | Timelags | Uptake | Time-variant? |
| HEC-RAS  (Gain et al. 2015) | RB | Hydrodynamic | N | P (early warning system) | Inst | R | V (flood depth-duration-damage) | S, H based on lead time and knowledge uptake | None | Uniformcoverage | No |
| LIS-Flood (Alfieri et al, 2016) | C | rainfall-runoff-routing model | N | P (improving defences; reducing peak flows, reducing vulnerability; relocation) | A | A, R | I (over-topping) | None | None | No | No |
| SWAP-WSBM (Droogers, 2004) | RB | 1D soil model + river basin water accounting model | N | A (irrigation depth; irrigated crop area) | A | A | E (increased yield variability) | B (water availability) | None | No (uniform) | No |
| WEAP |  |  |  |  |  |  |  |  |  |  |  |
| MPM -WEAP (Esteve et al. 2015) | RB | Farm optimisation + river basin water accounting model | N (WEAP); Yes (MPM) | P (tariffs; EFlows) + A (cropping choice) | Inst + Ind (representative farms) | A (tariffs; EFlows) + R (cropping) | E (optimising farm utility) | B (water allocation), H (labour) | None | Yes (farm type specific) | Yes (due to changing water allocation and crop yields) |
| Joyce et al (2011) | RB | Water accounting model | Y (logit equations programmed into WEAP) | P (irrigation efficiency) + A (cropping change) | A | A / R depending on adaptation | E (annual water resource status) | B (crop change) | No | No (but trend in uniform effectiveness) | Yes (maximum efficiency uptake by 2050; annual water resource status) |
| Mehta et al (2013) | RB | Water accounting model | N | A (irrigation technology improvement; cropping change) | A | A | None | No | No | No (but trend in effectiveness) | No- only as function of trends in adaptation scenarios (cropping; irrigation efficiency) |
| Purkey et al (2008) | RB | Water accounting model | N | P (irrigation efficiency) + A (cropping change) | A | A / R depending on adaptation | E (annual water resource status) | B (water allocation) | N | No (uniform) | No- only as climate change affects water resource |
| Rochdane et al (2012) | RB | Water accounting model | N | P (irrigation efficiency + domestic water saving) | A | A | E (unmet demand) | No | No | No (Maximum 50% uptake of drip irrigation; 100% for domestic) | No- only as function of trends in uptake and per-capita water saving |
| Santikayasa et al. (2014) | RB | Water accounting model | N | P (Irrigation area; crop intensity) | A | A | E (crop production) | B (water availability) | No | No (Uniform) | Only as function of any trends in adaptation scenarios |
| Vonk et al. (2014) |  | Water accounting model + external Metaheuristic Algorithm optimisation | Y | A (reservoir operating rules) | Inst | R | E (hydrpower and water supply) | B (flood control) | No | Complete No | No - only between climate and socioeconomic scenarios |
| Bhave et al. (2014a and 2014b) | RB | Water accounting model | N | P (forest cover, check dams) | A | A | E (streasmflow) | No (adaptation predefined); | No | No (Predefined and constant) | No |
| Bhave et al. (2016) | RB | Water accounting model | N | A (cropping pattern)+ P (forest cover, check dams, ponds and WW reuse) | A | A | E (streamflow) | No (adaptation predefined); B (water availability for cropping) | No | No (specified WW re-use in major towns only) | Only as function of trends in WW reuse |
| Bonelli et al 2014 | RB | Water accounting model | N | P (water rights, reservoir management; urban water efficiency) | Inst and A | R | I (maintain performance metric levels) | B (water availability) | No | No | Only due to population change |
| Lempert & Groves (2010) | RB | Water accounting model |  | P (increased WUE; greywater recycling; groundwater replenishment) | A | R | I (supply-demand surplus) | F (selection based on cost) | 5 year intervals | No (Uniform) | Y (adaptive strategy) |
| WaterGAP |  |  |  |  |  |  |  |  |  |  |  |
| LandSHIFT + WaterGAP Schaldach et al. (2012) | C | Landuse model + Process-based Hydrology & water use model | Y (LandSHIFT) | A (irrigated area) | A | R | I (irrigated crop production) | B | No | Yes (suitability-based) | No |
| Wimmer et al (2014) | C | Process-based Hydrology & water use model | N | P (Water allocation rules) | Inst | A | E (water over-exploitation) | B (water availability) | No | No | No |
| SWIM |  |  |  |  |  |  |  |  |  |  |  |
| Huang et al 2013 | RB | Ecohydrological process-based river basin model | Y (crop harvesting/planting) | A (planting and harvest dates) | A | R | E (harvest index( | No | No | No | No |
| Huang et al 2015 | RB | Ecohydrological process-based river basin model | N | A (irrigation area; changing crops) + P (channel lining) | A | A | None | No | No | 100% | No |
| Krysonova et al (2015) | RB | Ecohydrological process-based river basin model | N | P (reservoir construction) | Inst | A | None | No | No | N/A | No |
| VIC  (Zhou and Guo, 2013) | RB | Distributed macro-scale hydrological model + optimisation module | Y | P (reservoir release rules) | Inst (dam operator) | R | E (optimisation of flood control, water supply and Eflows) | M (reservoir and hydropower limits) | No | N/A | Yes (varies in response to scenario flow regimes) |
| SWAT |  |  |  |  |  |  |  |  |  |  |  |
| Carvalho-Santos et al. 2016 | RB | Semi-distributed physically-based model | N | A (landcover change) | A | A | None defined | No | No | No (uniform) | No (defined adaptation scenarios) |
| Chiang et al (2012) | RB | Semi-distributed physically-based model | N | P (grazing management, buffer strips and nutrient management) | A | A | E (water quality) | No | No | No (uniform) | No |
| Lakshmanan et al (2011) | RB | Semi-distributed physically-based model | N | A (alternative rice cultivation systems | A | R | E (rice production) | None | None | No (uniform) | No |
| Shin et al (2014) | RB | Semi-distributed physically-based model + LOGIT forest community model | N | A (natural forest vegetation community change) | N/A | R | E (forest community climate suitability | B | No | No (LOGIT suitability) | No (scenario conditions change but adaptation process constant) |
| Mango et al (2011) | RB | Semi-distributed physically-based model | N | A (deforestation to agriculture or grassland) | A | R | None defined | B (landuse) | No | No (uniform) | No |
| Walters & Babbar-Sebens (2016) | RB | Semi-distributed physically-based model | N | P (wetland re-creation) | A | R | E (peak flows) | B (suitable locations) | No | No (all sites) | No |
| SWAT + VenSim (Gies et al. 2014) | N | Systems dynamic model + SWAT | N | P (hydraulic infrastructure; agroforestry; drip irrigation) | A | A | E (water availability and food production) | B (water availability) and F (not constrained by F, but used to assess NPV) | No | Varied between options | No |
| CLUE + SWAT Mehdi et al. (2015) | RB | Land allocation model + SWAT | N | P (landuse management portfolios) | A | R | E (water quality) | B (landuse) | No | Variable (random or based on location or crop) | No (scenario conditions change but adaptation process constant) |
| MODIM + SWAT (Vaghefi et al 2015) |  | Water allocation DSS+ SWAT | N | P (wheat area) | A | R | E (food production; hydropower) | B (water allocation) | No | No (uniform) | No |
| GR4J Collet el al (2015) | RB | Semi-lumped rainfall-runoff model (GR4J) + unnamed dam management model | N | P (domestic water saving; water import) | A | R | I (reduced level of sustainability) | None | No | No (but spatially variable water savings & imports calculated) | No (scenario conditions change but adaptation process constant |
| CALVIN Connel-Buck et al. (2011) + Medellin-Azuara et al. (2008) | RB | economic-engineering optimization model | Y | A (cropping patterns) and P (reservoir operations & groundwater use) | A | R | I (minimise scarcity) | B/M [Physical and environmental constraints] | No | No (Uniform) | No (scenario conditions change but adaptation process constant |
| GR2M Girard et al. (2015 a & b) | RB | Semi-lumped rainfall-runoff model (GR2M) + ;least cost optimisation (LCRBOM) | Y | A (water efficiency improvements; behavioural change) / P (irrigation technologies, leakage control, desalination, groundwater) | A | R | I (meeting demand & Eflow) | S / H / F /B (feasibility; capability & acceptability; cost & effectiveness) | No | No (but spatial variability in optimum selection) | N |
| Unnamed Hayashi et al (2013) | G | Simple modular (agro-landuse model + water supply-demand) | N | A/P (cropping varieties & timing; irrigation efficiency; use of reclaimed water) | A | R | E (water stress) | M (limits to irrigation efficiency improvements and reclaimed water use in timestep) | No | No (but confined to water stressed basins) | No (scenario conditions change but adaptation process constant |
| InVEST + ELECTRE Kumar et al (2016) | RB | InVEST (hydrology) + out-ranking based decision making (ELECTRE) | Y | P (water recycling, desalination, interbasin transfers; demand management) | A | R | I (meeting demand) | F (least-cost); B (environmental impact) | N | No | No (scenario conditions change but adaptation process constant) |
| WBalMo1Koch & Vogele (2009) | RB | Water resource management model | N | A (maintenance timing;) and P (power plant water intake; plant modification) | Inst | R | I (shortage due to water availability & temperature) | Selection based on F; M (options differ according to power station characteristics) | N | Yes (power station characteristics) | No (scenario conditions change but adaptation process constant) |
| OASIS Sauchyn et al. (2016) | RB | Hydrologic mass balance model | N | P (Changes to existing and new infrastructure capacity & operation) | A | R + A (forecast-based) | I (water shortage) | B (minimum river flows) | None | No | No |
| CLIRUN-II Ward et al. (2010) | G | Rainfall-runoff model + cost curves | N | P (increased reservoir yield or alternative sources [recycling, rainwater harvesting, desalination]) | A | R | I (meeting Industrial and agricultural water demand) | F (cost-curves for reservoir costruction) | No | No | No (scenario conditions change but adaptation process constant) |
| Unnamed Zhang and Lin (2015) | C | Empirical model | N | A (irrigation area) | A | R | E (change in irrigation need) | None | None | No (unform) | No |

**References for Table SM2**

Alfieri L, Feyen L, Di Baldassarre G (2016). Increasing flood risk under climate change: a pan-European assessment of the benefits of four adaptation strategies. Clim Change 136: 507-521. DOI: 10.1007/s10584-016-1641-1

Bhave AG, Mishra A, Raghuwanshi NS (2014). A combined bottom-up and top-down approach for assessment of climate change adaptation options. J Hydrol 518: 150-161. DOI: 10.1016/j.jhydrol.2013.08.039

Bhave AG, Mishra A, Raghuwanshi NS (2014). Evaluation of hydrological effect of stakeholder prioritized climate change adaptation options based on multi-model regional climate projections. Clim Change 123: 225-239. DOI: 10.1007/s10584-014-1061-z

Bhave AG, Mittal N, Mishra A, Raghuwanshi NS (2016). Integrated Assessment of no-Regret Climate Change Adaptation Options for Reservoir Catchment and Command Areas. Water Res manage 30: 1001-1018. DOI: 10.1007/s11269-015-1207-4

Bonelli S, Vicuna S, Meza FJ, Gironas J, Barton J (2014). Incorporating climate change adaptation strategies in urban water supply planning: the case of central Chile. J Water Clim Change 5: 357-376. DOI: 10.2166/wcc.2014.037

Carvalho-Santos C, Nunes JP, Monteiro AT, Hein L, Honrado JP (2016). Assessing the effects of land cover and future climate conditions on the provision of hydrological services in a medium-sized watershed of Portugal. Hydrolog Proc 30: 720-738. DOI: 10.1002/hyp.10621

Chiang L-C, Chaubey I, Hong N-M, Lin Y-P, Huang T (2012). Implementation of BMP Strategies for Adaptation to Climate Change and Land Use Change in a Pasture-Dominated Watershed. Int J Environ Res Public Health 9: 3654-3684. DOI: 10.3390/ijerph9103654

Collet L, Ruelland D, Estupina VD, Dezetter A, Servat E (2015). Water supply sustainability and adaptation strategies under anthropogenic and climatic changes of a meso-scale Mediterranean catchment. Sci Total Environ 536: 589-602. DOI: 10.1016/j.scitotenv.2015.07.093

Connell-Buck CR, Medellin-Azuara J, Lund JR, Madani K (2011). Adapting California's water system to warm vs. dry climates. Clim Change 109: 133-149. DOI: 10.1007/s10584-011-0302-7

Droogers P (2004). Adaptation to climate change to enhance food security and preserve environmental quality: example for southern Sri Lanka. Agr Water Manage 66: 15-33. DOI: 10.1016/j.agwat.2003.09.005

Esteve P, Varela-Ortega C, Blanco-Gutierrez I, Downing TE (2015). A hydro-economic model for the assessment of climate change impacts and adaptation in irrigated agriculture. Ecolog Economics 120: 49-58. DOI: 10.1016/j.ecolecon.2015.09.017

Gain AK, Mojtahed V, Biscaro C, Balbi S, Guipponi C (2015). An integrated approach of flood risk assessment in the eastern part of Dhaka City. Nat Hazards 79: 1499-1530. DOI: 10.1007/s11069-015-1911-7

Gies L, Agusdinata DB, Merwade V (2014). Drought adaptation policy development and assessment in East Africa using hydrologic and system dynamics modeling. Nat Hazards 74: 789-813. DOI: 10.1007/s11069-014-1216-2

Girard C, Pulido-Velazquez M Rinaudo J-D, Page C, Caballero Y (2015). Integrating top-down and bottom-up approaches to design global change adaptation at the river basin scale. Global Environ Change 34: 132-146. DOI: 10.1016/j.gloenvcha.2015.07.002

Girard C, Rinaudo J-D, Pulido-Velazquez M, Caballero Y (2015). An interdisciplinary modelling framework for selecting adaptation measures at the river basin scale in a global change scenario. Environ Model Software 69: 42-54. DOI: 10.1016/j.envsoft.2015.02.023

Hayashi A, Akimoto K, Tomoda T, Kii M (2013). Global evaluation of the effects of agriculture and water management adaptations on the water-stressed population. Mitig Adapt Strat Global Change 18: 591-618. DOI: 10.1007/s11027-012-9377-3

Huang S, Hattermann FF, Krysanova V, Bronstert (2013). Projections of climate change impacts on river flood conditions in Germany by combining three different RCMs with a regional eco-hydrological model. Clim Change 116: 631-663. DOI: 10.1007/s10584-012-0586-2

Huang S, Hattermann FF, Zhai J, Su B (2015). Impact of Intensive Irrigation Activities on River Discharge Under Agricultural Scenarios in the Semi-Arid Aksu River Basin, Northwest China. Water Resour Manage 29: 945-959. DOI: 10.1007/s11269-014-0853-2

Joyce BA, Mehta VK, Purkey DR, Dale LL, Hanemann M (2011). Modifying agricultural water management to adapt to climate change in California's central valley. Clim Change 109: 299-316. DOI: 10.1007/s10584-011-0335-y

Koch H, Voegele S (2009). Dynamic modelling of water demand, water availability and adaptation strategies for power plants to global change. Ecolog Economics 68: 2031-2039. DOI: 10.1016/j.ecolecon.2009.02.015

Krysanova V, Hattermann F, Huang S, Hesse C, Vetter T, Liersch S, Koch H, Kundewicz ZW (2015). Modelling climate and land-use change impacts with SWIM: lessons learnt from multiple applications. Hydrol Sci J 60(4): 606-635. DOI 10.1080/02626667.2014.925560

Kumar V, Del Vasto-Terrientes L, Valls A, Schuhmacher M (2016). Adaptation strategies for water supply management in a drought prone Mediterranean river basin: Application of outranking method. Sci Total Environ 540: 344-357. DOI: 10.1016/j.scitotenv.2015.06.062

Lakshmanan A, Geethalakshmi V, Rajalakshmi D, Bhuvaneswari K, Srinivasan R, Sridhar G, Sekhar NU, Annamalai H (2011). Climate change adaptation strategies in the Bhavani basin using the SWAT model. Appl Engineer Agric 27: 887-893.

Lempert RJ, Groves, DG (2010). Identifying and evaluating robust adaptive policy responses to climate change for water management agencies in the American west. Technological Forecasting And Social Change 77: 960-974. DOI: 10.1016/j.techfore.2010.04.007

Mango LM, Melesse AM, McClain ME, Gann D, Setegn SG (2011). Land use and climate change impacts on the hydrology of the upper Mara River Basin, Kenya: results of a modeling study to support better resource management. Hydrol Earth Syst Sci 15: 2245-2258. DOI: 10.5194/hess-15-2245-2011

Medellin-Azuara J, Harou JJ, Olivares MA, Madani K, Lund JR, Howitt RE, Tanaka SK, Jenkins MW, Zhu T (2008). Adaptability and adaptations of California's water supply system to dry climate warming. Clim Change 87: S75-S90. DOI: 10.1007/s10584-007-9355-z

Mehdi B, Lehner B, Gombault C, Michaud A, Beaudin I, Sottile M-F, Blondlot A (2015). Simulated impacts of climate change and agricultural land use change on surface water quality with and without adaptation management strategies. Agric Ecosyst Environ 213: 47-60. DOI: 10.1016/j.agee.2015.07.019

Mehta VK, Haden VR, Joyce BA, Purkey DR, Jackson LE (2013). Irrigation demand and supply, given projections of climate and land-use change, in Yolo County, California. Agric Water Manage 117: 70-82. DOI: 10.1016/j.agwat.2012.10.021

Purkey DR, Joyce B, Vicuna S, Hanemann MW, Dale LL, Yates D, Dracup JA (2008). Robust analysis of future climate change impacts on water for agriculture and other sectors: a case study in the Sacramento Valley. Clim Change 87: S109-S122. DOI: 10.1007/s10584-007-9375-8

Rochdane S, Reichert B, Messouli M, Babqiqi A, Khebiza MY (2012). Climate Change Impacts on Water Supply and Demand in Rheraya Watershed (Morocco), with Potential Adaptation Strategies. Water 4: 28-44. DOI: 10.3390/w4010028

Santikayasa IP, Babel MS, Shrestha S, Jourdain D, Clemente RS (2014). Evaluation of water use sustainability under future climate and irrigation management scenarios in Citarum River Basin, Indonesia. Int J Sustainable Development and World Ecology 21: 181-194. DOI: 10.1080/13504509.2014.884023

Sauchyn DJ, St-Jacques J-M, Barrow E, Nemeth, MW, MacDonald, RJ, Sheer, AMS, Sheer, DP (2016). Adaptive water resource planning in the South Saskatchewan river basin: use of scenarios of hydroclimatic variability and extremes. J American Water Resources Assoc 52: 222-240. DOI: 10.1111/1752-1688.12378

Schaldach Ru, Koch J, derBeek TA, Kynast E, Floerke M (2012). Current and future irrigation water requirements in pan-Europe: An integrated analysis of socio-economic and climate scenarios. Global and Planetary Change 94-95: 33-45. DOI: 10.1016/j.gloplacha.2012.06.004

Shin H-J, Park M-J, Hwang S-J, Park J-Y, Kim S-J (2014). Hydrologic impact of climate change with adaptation of vegetation community in a forest-dominant watershed. Paddy and Water Environ 12: S51-S63. DOI: 10.1007/s10333-014-0426-2

Vaghefi SA, Mousavi SJ, Abbaspour KC, Srinivasan R, Arnold JR (2015). Integration of hydrologic and water allocation models in basin-scale water resources management considering crop pattern and climate change: Karkheh River Basin in Iran. Reg Environ Change 15: 475-484. DOI: 10.1007/s10113-013-0573-9

Vonk E, Xu YP, Booij MJ, Zhang X, Augustijn DCM (2014). Adapting Multireservoir Operation to Shifting Patterns of Water Supply and Demand. Water Res Manage 28: 625-643. DOI: 10.1007/s11269-013-0499-5

Walters KM, Babbar-Sebens M (2016). Using climate change scenarios to evaluate future effectiveness of potential wetlands in mitigating high flows in a Midwestern US watershed. Ecolog Engineer. 89: 80-102. DOI: 10.1016/j.ecoleng.2016.01.014

Ward PJ, Strzepek KM, Pauw WP, Brander LM, Hughes GA, Aerts JCJH (2010). Partial costs of global climate change adaptation for the supply of raw industrial and municipal water: a methodology and application. Environ Res Letters 5: 44011 DOI: 10.1088/1748-9326/5/4/044011.

Wimmer F, Audsley E, Masly M, Savin C, Dunford R, Harrison PA, Schaldach R, Florke M (2014). Modelling the effects of cross-sectoral water allocation schemes in Europe. Clim Change 128: 229-244. DOI 10.1007/s10584-014-1161-9

Zhang T, Lin X, Rogers DH, Lamm FR (2015). Adaptation of Irrigation Infrastructure on Irrigation Demands under Future Drought in the United States. Earth Interactions 19: 1-16. DOI: 10.1175/EI-D-14-0035.1

Zhou Y, Gio S (2013). Incorporating ecological requirement into multipurpose reservoir operating rule curves for adaptation to climate change. J Hydrol 498: 153-164. DOI: 10.1016/j.jhydrol.2013.06.028

1. G = Global; C = Continental; N = national; RB = River Basin; F = Farm [↑](#footnote-ref-1)
2. A = Autonomous; P = Planned [↑](#footnote-ref-2)
3. Ind = Individual; Inst – Institution (formal or informal); G = Government, A= Aggregate (or Unspecified) [↑](#footnote-ref-3)
4. A = Anticipatory; R = Reactive [↑](#footnote-ref-4)
5. E = based on expert judgement/subjective; I = based on Impact trigger; V = based on vulnerability trigger [↑](#footnote-ref-5)
6. B = biophysical; S = Social; H = Human; F = financial; M = manufactured [↑](#footnote-ref-6)
7. G = Global; C = Continental; N = national; RB = River Basin; F = Farm [↑](#footnote-ref-7)
8. A = Autonomous; P = Planned [↑](#footnote-ref-8)
9. Ind = Individual; Inst – Institution (formal or informal); G = Government, A= Aggregate (or Unspecified) [↑](#footnote-ref-9)
10. A = Anticipatory; R = reactive [↑](#footnote-ref-10)
11. E = based on expert judgement/subjective; I = based on Impact trigger; V = based on vulnerability trigger [↑](#footnote-ref-11)
12. B = biophysical; S = Social; H = Human; F = financial; M = manufactured [↑](#footnote-ref-12)
